# “Lost between the interface of physical and mental health”: focus groups exploring liaison psychiatry staff's perception about working during the first wave of the COVID-19 pandemic in Birmingham and Solihull Mental Health Foundation Trust

**DOI:** 10.1192/bjo.2021.864

**Published:** 2021-06-18

**Authors:** Saima Jehanzeb, Muhammad Suleman, Ella Tumelty, Joanne Okusanya, Laxsan Karunanithy, Lucretia Thomas, Dhruba Bagchi, Mahnaz Hashmi

**Affiliations:** 1 Birmingham and Solihull Mental Health NHS Foundation Trust; 2 Birmingham Community Healthcare NHS Foundation Trust; 3 Medical School, College of Medical and Dental Sciences, University of Birmingham

## Abstract

**Aims:**

As the COVID-19 pandemic continues, increasing attention is being drawn to the welfare of healthcare providers who have endured many months of sustained exposure to the virus, disrupted working conditions and psychological stress. This project aimed to explore the subjective experiences of staff working in Liaison Psychiatry (LP) in the Birmingham and Solihull Mental Health Foundation Trust, (BSMHFT) during the first wave of the COVID-19 pandemic. These findings have been used to devise recommendations for subsequent waves.

**Method:**

Data collection occurred as part of a mixed method service evaluation project. We invited all clinical and non-clinical staff from LP departments across BSMHFT to participate in focus groups conducted via Microsoft Teams. The focus groups were video-recorded and facilitated by a moderator and an observer. Subsequent anonymised transcripts were coded and themes were generated by at least two evaluators, using thematic analysis.

**Result:**

The focus groups, which ranged from 21 to 69 minutes, involved consultants, junior doctors and nurses from four hospitals within BSMHFT. Six major themes emerged including an initial reduction in number yet increase in acuity of patients seen by LP, with some perception that this resulted from reduced face-to-face contact with community mental health services. A feeling that LP was lost at the interface between the physical and mental health trusts emerged as another theme. Uncertainty in adapting to unprecedented working conditions, for example, unclear guidance concerning the use of personal protective equipment, was also described alongside anxiety about contracting and transmitting SARS-Cov-2. Additionally, increased pressure was felt due to staff shortages and inadequate inter-departmental communication. Participants reported differential uptake of remote working, as well as conflicting views regarding the feasibility of remote assessments in LP.

**Conclusion:**

Liaison psychiatry staff within BSMHFT continued to provide a crucial service during the COVID-19 pandemic. Focus groups with thes staff indicate several recommendations for implementation within the Trust and provoke questions for future research. Due to the unique role that LP plays in providing mental health care within general hospitals, clear guidance for LP staff is key for effective service provision and supporting LP staff. Although used widely across community mental health services, the role of remote working in LP is contentious and requires further exploration. However, there are limitations to the use of focus groups and these findings may not fully represent the experiences of LP staff throughout BSMHFT. Different themes may have emerged through the use of anonymous questionnaires.

